# Hippurate of Soda in the Treatment of Lithæmia

**Published:** 1886-04

**Authors:** 


### Hippurate of Soda in the Treatment of Lithæmia.

Garrod has shown that the *hippurate of soda* increases in a marked manner the elimination of lithic acid from the system. Bon recommends the following formula to be used in the treatment of all affections characterized by an excess of uric acid:

℞ Sodii Hippurat, - - - ʒ i, gr. xv.

Lithic Carbonate, - - - gr. xxiv.

Glycerinæ, - - - - - ℥ iv.

Aq. Menth., - - - - ʒ viii.

M. Sig.: Tablespoonful four times a day.

Or the following:

℞ Sodii Hippurat, - - - ʒ iss.
Potassæ Chloratis, - - - - gr. xx.
Syr. Simp., - . - - ℥ i.
Aq. Menth., - - - - - ℥ vi.

M. Sig.: Tablespoonful four to six times a day.

*Il. Raccoglitore Med.*, No. vi., 1886.

